# Maternal Exposure to Air Pollution and Birth Outcomes

**DOI:** 10.1289/ehp.1002564

**Published:** 2011-01-06

**Authors:** Ebba Malmqvist, Anna Rignell-Hydbom, Håkan Tinnerberg, Jonas Björk, Emilie Stroh, Kristina Jakobsson, Ralf Rittner, Lars Rylander

**Affiliations:** Division of Occupational and Environmental Medicine, Lund University, Lund, Sweden

**Keywords:** air pollution, fetal growth restriction, LBW, low birth weight, NO_x_, pregnancy, preterm births, preterm delivery, SGA

## Abstract

**Background:**

The knowledge about air pollution effects on birth weight, prematurity, and small for gestational age (SGA) in low-exposure areas is insufficient.

**Objectives:**

The aim of this birth cohort study was to investigate whether low-level exposure to air pollution was associated with prematurity and fetal growth and whether there are sex-specific effects.

**Method:**

We combined high-quality registry information on 81,110 births with individually modeled exposure data at residence for nitrogen oxides (NO_x_) and proximity to roads with differing traffic density. The data were analyzed by logistic and linear regression with and without potential confounders.

**Results:**

We observed an increased risk for babies being SGA when we compared highest and lowest NO_x_ quartiles, adjusting for maternal age, smoking, sex, and year of birth. After additional adjustment for maternal country of origin and parity (which were highly intercorrelated), the increase was no longer statistically significant. However, in subgroup analyses when we compared highest and lowest NO_x_ quartiles we still observed an increased risk for SGA for girls [odds ratio (OR) = 1.12; 95% confidence interval (CI), 1.01–1.24); we also observed increased risk among mothers who had not changed residency during pregnancy (OR = 1.09; 95% CI, 1.01–1.18). The confounders with the greatest impact on SGA were parity and country of origin. Concerning prematurity, the prevalence was lower in the three higher NO_x_ exposure quartiles compared with the lowest category.

**Conclusion:**

For future studies on air pollution effects on birth outcomes, careful control of confounding is crucial.

Intrauterine growth restriction—which manifests as low birth weight (LBW), “small for gestational age” (SGA), and preterm birth (PTB)—not only is a predictor for infant mortality and morbidity (Petrini et al. 2004) but also has implications from a life-course perspective. A wealth of epidemiologic evidence has provided a convincing link between a suboptimal gestational environment and an increased risk for adult onset of metabolic as well as nonmetabolic diseases (Joss-Moore and Lane 2009). Knowledge of the underlying factors for growth restriction and preterm delivery is therefore of great concern. Intrauterine growth restriction may result from disturbances to placental blood flow, poor maternal nutrition, or maternal exposure to toxicants. Several studies have shown, although not consistently, that air pollution is a risk factor for PTBs and altered fetal growth, especially in high-exposure areas (Bobak 2000; Bobak and Leon 1999; Ha et al. 2001; Maisonet et al. 2004; Ritz and Yu 1999; Slama et al. 2009; Wilhelm and Ritz 2003).

It has, however, been clearly stated that future research on air pollution and birth outcomes must confirm that observed air pollution effects on birth weights, prematurity, and SGA are genuine, causal, and not attributable to confounding factors and to investigate whether the effect also remains in low-exposure areas (Dugandzic et al. 2006; Jedrychowski et al. 2009; Liu et al. 2003; Maroziene and Grazuleviciene 2002). Interestingly, it has been suggested, in some studies, that the sex of an infant can play an important role in sensitivity to air pollution and related birth outcomes (Ghosh et al. 2007; Jedrychowski et al. 2009).

There have been shortcomings in some of the previous studies with regard to exposure assessments. The exposure data are often based on stationary air pollution monitors, which are not considered to provide optimal resolution when attributed to individual exposures (Slama et al. 2008). When performed by personal monitoring, monitoring time is shorter than the attributed exposure period and thus does not reflect exposure throughout the pregnancy. Moreover, in such studies the numbers of participants are inevitably low, which affects the statistical power. More accurate exposure assessment methods have been discussed (Slama et al. 2008), identifying exposure models with high spatial and temporal resolutions or simple source models using distance-weighted traffic density as promising tools. In Scania, Sweden, unique personal identity codes, geocoded information on each individual’s residence, an extensive emission database, road traffic data, and high-quality information from the Medical Birth Registers can be used, using geographic information systems, for linkage of data.

The aim of our birth cohort study was to investigate whether low-level exposure to air pollution was associated with prematurity and fetal growth and whether there are sex-specific effects.

## Materials and Methods

### Study area and population

**Figure 1:**
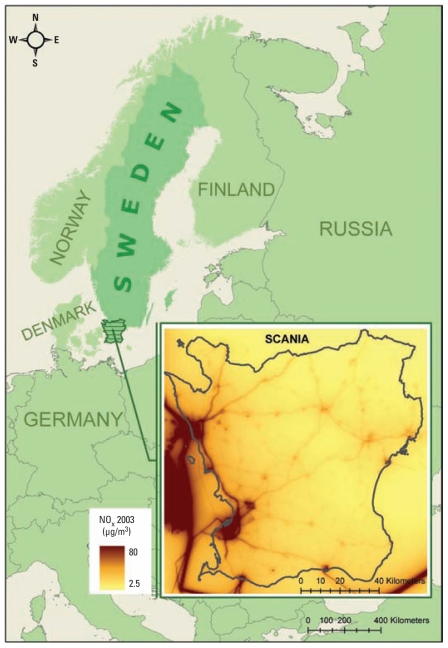
Study area and annual mean levels of NO_x_ for 2003.

Scania (Skåne) is the southernmost county in Sweden (Figure 1), covering around 11,350 km^2^ (~ 2% of the total area of Sweden). It is one of the most densely populated areas of the country, with about 1.1 million people (~ 11% of the total Swedish population). Compared with other areas of Sweden, the level of air pollutants in the western part of Scania (where most people live) can be high because of road transportation to and from the European continent and a considerable amount of cargo shipping and ferry transport along the coast. However, air pollutant levels are generally well below the present World Health Organization air quality guideline (World Health Organization 2006) and low in a European perspective (Sjöberg et al. 2006).

Data concerning all deliveries in Scania during the period 1999–2005 were obtained from a regional birth registry, Perinatal Revision Syd, and from the national Swedish Medical Birth Registry containing background characteristics such as maternal age, smoking habits, weight, country of origin, and parity, as well as delivery outcome. The Swedish Medical Birth Registry includes almost every infant (98–99%) born in Sweden (Socialstyrelsen 2002). Of a total of 84,039 registered births for the period 1999–2005, we excluded 2,929 births that were not registered as singleton births. The total number of births included in the analyses depends on outcome and exposure measure (Table 1).

### Outcome measures

The following outcome measures were used: *a*) birth weight as a continuous variable; *b*) LBW, defined as birth weight < 2,500 g according to the *International Classification of Diseases, 10th Revision* (ICD-10) (World Health Organization 1993), codes P07.0–P0.73; *c*) PTB, defined as < 37 weeks of gestation according to ICD-10 codes P07.0–P07.3 (> 98% of the gestational length estimations in the Swedish Medical Birth Registry are based on routinely made early ultrasound examination, and the remaining gestational length estimations are based on information on last menstrual period); and *d*) SGA, defined as a birth weight < 10th percentile compared with the birth weight distribution for the same gestational week and sex according to ICD-10 codes P07.0–P07.3. The cutoff limits, that is, 10th percentiles, were based on data for the total Swedish population during the period concerned; data were obtained from the Swedish Medical Birth Registry.

When analyzing for LBW, we excluded 295 births with missing birth weight and 463 births with invalid data operationally defined as a birth weight < 500 g or > 6,500 g. As for PTB, we excluded 352 births with a missing gestational length and 356 births with invalid gestational length (operationally defined as < 20 weeks or > 44 weeks). In the SGA analysis, valid data on both gestational length and birth weight were required.

### Exposure assessments

#### Linkage of databases

Each resident in Sweden has a unique 10-digit personal identification code, which can be linked to the spatial coordinates of their place of residence (yearly updated). Individuals are positioned according to the center coordinate of their place of residence. Mother and child data from the birth registries can thus be linked to geocoded environmental databases.

#### Modeled nitrogen oxides exposure

We used an emission database (EDB) for nitrogen oxides (NO_x_) in Scania, which contains information on emissions from approximately 24,000 sources (Stroh et al. 2005). Most of these sources are line sources corresponding to road traffic, shipping, and railroads. Point sources include industries and larger energy and heat producers. Area sources included—for example, aviation and emissions from small-scale heating and construction machinery. Because emissions from eastern Denmark are quite high and westerly winds are dominant, emissions from eastern Denmark have also been added into the EDB. The EDB was combined with a modified Gaussian dispersion model, AERMOD (U.S. Environmental Protection Agency 2004), used for dispersion calculations. AERMOD is a flat two-dimensional model taking the height of the emission source as well as meteorology into account. However, the model does not adjust for effects of street canyons or other terrain effects. To account for long-range transboundary air pollution, we added a background level of 2.5 μg/m^3^ into the model corresponding to the yearly mean from a remote background monitor station. The modeled concentration in the EDB has been plotted against measured concentration in order to validate the model, and the *R*^2^ was 0.69 (Stroh et al. 2005).

Concentrations of NO_x_ were modeled as hourly means in each cell using a spatial resolution of 500 × 500 m. These hourly means were aggregated to individually calculated gestational monthly and trimester means (months 1–3 of pregnancy, months 4–6, and month 7 to delivery). The spatial resolution of 500 × 500 m in this EDB has been shown to be appropriate, concerning accuracy, when studying aggregated monthly means (Stroh et al. 2007).

Because of migration in and out of the county, we lacked information on trimester-specific NO_x_ exposure for a small proportion of births (first trimester, 5,201 births; second trimester, 4,756 births; third trimester, 4,585 births), although they were included in the trimesters when we had their exposure data.

We used a categorical classification of NO_x_ by dividing the exposure levels into quartiles based on overall data from the first trimester, giving the following cutoff values: *a*) 2.5–8.9 μg/m^3^ (mean, 6.8 μg/m^3^), the reference category; *b*) 9.0–14.1 μg/m^3^ (mean, 11.4 μg/m^3^); *c*) 14.2–22.6 μg/m^3^ (mean, 18.2 μg/m^3^); and *d*) > 22.7 μg/m^3^ (mean, 29.6 μg/m^3^).

#### Traffic density

The geocoded location of the residency was also used for calculation of the distance between the mother’s residence and roads with different traffic density, which was used as a proxy for pollution generated by road traffic. Traffic density was obtained from the Swedish National Road Database (Vägverket 2010). Each individual was assigned the road with the heaviest traffic density (annual 24-hr mean level), within a radius of 100 m from their residence early in pregnancy. We excluded 5,536 births with missing traffic density exposure because of migration.

The traffic density variable was categorized as *a*) no road within 100 m from place of residence, the reference category; *b*) road with < 2 cars/min; *c*) road with 2–5 cars/min; *d*) road with 6–10 cars/min; and *e*) road with > 10 cars/min.

### Background characteristics and potential confounders

Tables 1 and 2 list background characteristics for mothers and children in relation to birth outcomes and NO_x_-exposure categories.

Based on previous knowledge, we considered the following variables as potential confounders: maternal age (four categories: < 25, 25–30, 30–35, and > 35 years), sex (already included in the SGA definition), smoking reported during early pregnancy (four categories: nonsmoker, smoking 1–9 cigarettes/day, smoking ≥ 10 cigarettes/day, and missing smoking information), and parity [three categories: parity 1 (first-born children), parity 2 (mother’s second pregnancy), and parity ≥ 3 (mother’s third or more pregnancy)] (Källén 1988). We also included birth year of the child (1-year categories) to adjust for calendar year effects.

**Figure 2:**
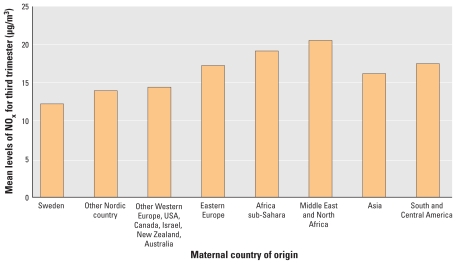
Mean level of NO_x_ (μg/m^3^) by maternal country of origin (without added background level of NO_x_).

Country of origin was available for almost all mothers and was used as an indicator of sociodemographic status (classified into eight categories: Sweden; other Nordic countries; other Western European countries, United States, Australia, Canada, New Zealand, and Israel; Eastern European countries; Africa sub-Saharan countries; North African and Middle Eastern countries; Asian countries; and South and Central American countries). Figure 2 demonstrates the relationship between NO_x_ exposure and maternal country of origin.

### Statistical analysis

We analyzed the dichotomous outcome variables (LBW, PTB, and SGA) using logistic regression models. We used linear regression models for birth weight as a continuous outcome variable with both unadjusted and adjusted analyses. Recent studies have suggested that maternal country of origin is an important confounder (Moore et al. 2009; Rasmussen et al. 1995; Zeka et al. 2008). In our study there was a strong correlation between parity and country of origin (distribution percentage for parity: in the Swedish population, parity 1, 51%; parity 2, 34%; parity ≥ 3, 16%; for any other country group within Sweden, 26%, 23%, and 51%, respectively). Thus, we performed the following analyses for each outcome: *a*) unadjusted, with no confounders included; *b*) with a minimum set of confounders (maternal age and smoking, and birth year and sex of child); *c*) with the minimum set of confounders and parity; *d*) with the minimum set of confounders and maternal country of origin; *e*) with the minimum set of confounders plus parity and maternal country of origin; *f*) a subanalysis including only first-born children, with the minimum set of confounders and maternal country of origin; *g*) a subanalysis including only mothers of Nordic origin, with the minimum set of confounders and parity; and *h*) a subanalysis including only mothers who had not moved during pregnancy (because the geocoded maternal place of residence is updated yearly, there may be some misclassification of mothers who have moved during pregnancy), with the minimum set of confounders, parity, and country of origin.

To understand why maternal country of origin was of importance for the SGA results, despite relatively large fractions of missing data, we performed an additional analysis where we added maternal height (10% missing) and body mass index (25.7% missing) to the model, to see whether maternal body stature explained the findings. We also explored information on employment status (20% missing), as a marker of socioeconomic situation and social anchorage, by adding it to the models.

To explore if sex was an effect modifier, we performed stratified analyses. Moreover, we included an interaction term (exposure × sex) based on Wald’s method in the adjusted models. For LBW, a subgroup analysis including only term births was performed, to address the potentially distinct etiology of LBW due to growth restriction. To assess the robustness of our results, we also performed subgroup analyses, including only Nordic origin mothers who had not moved during pregnancy, both for first-born children only and for all children.

Exposure data for the different trimesters were analyzed separately. Hence, the findings were similar for all trimesters, and we therefore present only results from the third trimester.

We used PASW Statistics (version 18; SPSS Inc. 2009).

## Results

The mean exposure level of NO_x_ in the study area was 16.4 μg/m^3^ during 1999–2005. During the same period, 2,863 babies were born with LBW (3.5% of all singleton births), 4,690 babies were born PTB (5.8% of all singleton births), and 7,821 babies were classified as SGA (9.8% of all singleton births).

### Birth weight

Neither individually modeled NO_x_ levels nor traffic density was associated with the risk of having a baby with LBW, when we included all individuals in the analyses (Table 3). We observed no interaction between sex and exposure (sex × NO_x_ interaction, *p* = 0.49; sex × traffic density interaction, *p* = 0.71).

For birth weight as a continuous variable, we observed a statistically significant association with modeled NO_x_ in the unadjusted analysis but not in the fully adjusted analyses. The birth weight loss, for the highest quartile of NO_x_ compared with the lowest, was 75 g [95% confidence interval (CI), 64–85 g] in the unadjusted analysis and 11 g (95% CI, 0–23 g) in the fully adjusted analysis. For traffic density, comparing the category with > 10 cars/min with the lowest category, the birth weight loss was 71 g (95% CI, 52–89 g) in the unadjusted analysis and 12 g (95% CI, 6–30 g) in the adjusted analysis. As for LBW, we observed no interaction between sex and exposure (sex × NO_x_ interaction, Wald *p* = 0.50; sex × traffic density interaction, Wald *p* = 0.66).

The pattern was very similar in the subgroup analyses, when we included only mothers of Nordic origin or mothers who had not moved during pregnancy (data not shown). The effect estimates did not change substantively when we restricted our analyses to include only babies that were born at term. We observed a statistically significant association between individually modeled NO_x_ and LBW when we included only first-born children; for the highest exposure quartile of NO_x_ compared with the lowest, the risk was reduced, with an odds ratio (OR) of 0.79 (95% CI, 0.68–0.93), adjusted for all confounders (data not shown).

### PTB

The risk of PTB was lower in the three higher NO_x_ exposure quartiles compared with the reference category (Table 3). The unadjusted and adjusted ORs varied between 0.85 and 0.91. For traffic density, we observed no statistically significant associations, but we observed a tendency to a lower risk of PTB in the category > 10 cars/min compared with the reference category (OR = 0.88; 95% CI, 0.76–1.02) adjusted for all confounders. We observed no interaction between sex and exposure (sex × NO_x_ interaction, Wald *p* = 0.80; sex × traffic density interaction, Wald *p* = 0.55). The results were largely identical for the analyses where we included only mothers of Nordic origin, only first-born children, or only mothers who had not changed residence during pregnancy (data not shown).

### SGA

**Figure 3:**
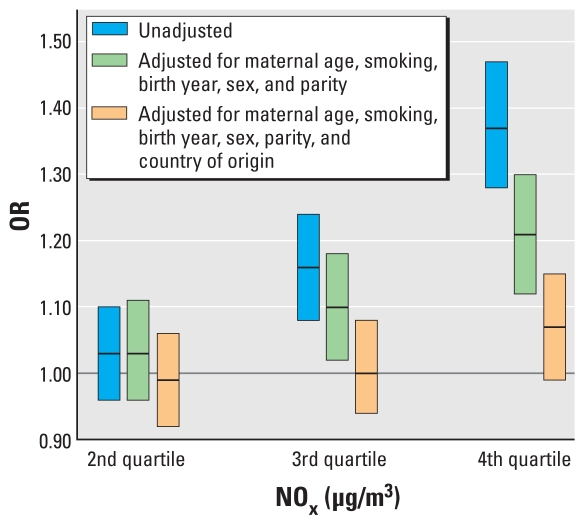
ORs (95% CIs) for a mother having an SGA baby for NO_x_ levels in the second quartile (9.0–14.1 μg/m^3^), third quartile (14.2–22.6 μg/m^3^), or fourth quartile (> 22.7 μg/m^3^) compared with the first quartile (2.5–8.9 μg/m^3^).

In the unadjusted analyses, we observed statistically significant associations between SGA and both NO_x_ and traffic density. For the highest exposure quartile of NO_x_ compared with the lowest category, the OR was 1.37 (95% CI, 1.28–1.47). For traffic density, comparing the category with > 10 cars/min with the reference category, the OR was 1.26 (95% CI, 1.14–1.40). After adjusting for the minimum set of confounders, the ORs were 1.29 (95% CI, 1.20–1.38) and 1.12 (95% CI, 1.01–1.24), respectively. Adding country of origin and removing parity from the model gave ORs of 1.21 (95% CI, 1.12–1.30) and 1.19 (95% CI, 1.07–1.33), respectively (for more details, see Table 3, Figure 3). After adjusting for all confounders, no statistically significant results remained, with ORs of 1.07 (95% CI, 0.99–1.15) and 1.04 (95% CI, 0.93–1.15). However, in the subanalyses the results were still statistically significant for NO_x_ exposure for mothers who had not changed their residency during pregnancy, with an OR of 1.09 (95% CI, 1.01–1.18). The effect estimates went in the direction of higher ORs among baby girls (OR = 1.12; 95% CI, 1.01–1.24) in the highest exposure category. However, we observed no statistically significant interactions between sex and exposure (sex × NO_x_ interaction, *p* = 0.09; sex × traffic density interaction, *p* = 0.23).

We observed no statistically significant associations in the adjusted analyses when we included only mothers of Nordic origin. The findings were similar when restricted to only first-born children or to mothers that had not changed their residency during pregnancy (data not shown), but not if we included only Nordic mothers who had not changed residency during pregnancy or if those mothers were restricted to parity 1. Additional analyses where we included maternal height, body mass index, and employment status in the models did not alter the results (data not shown).

## Discussion

Results from this large population-based study suggest that although we did find an association between SGA babies and maternal exposure to air pollution in unadjusted or partly adjusted analyses, those results did not remain statistically significant after adjusting for all potential confounders. This stated, a small exception was that we observed a statistically significant, but fairly small, risk for SGA for baby girls and in a subanalysis including only mothers who had not changed residency during pregnancy.

In the present study, country of origin and parity were strong confounders. When controlling for country of origin and parity separately, we still had statistically significant results. This may be attributable to the strong correlation between parity and country of origin. Previous studies in our study area have shown that the populations of non-Nordic origin tend to live in more polluted areas (Chaix et al. 2006; Stroh et al. 2005). The effect of country of origin on the risk of having a baby with reduced birth weight is well known (Moore et al. 2009; Rasmussen et al. 1995; Zeka et al. 2008) and has also been demonstrated in Malmö, the largest city in Scania (Dejin-Karlsson and Östergren 2004). In the previous Malmö study, the main explanation did not seem to be maternal physical size, but rather maternal psychosocial factors such as low social anchorage (Dejin-Karlsson and Östergren 2004). Hence, effects from the mother’s country of origin—independently if the causality behind is due to maternal stature or social anchorage—and exposure to air pollution need to be separated. In our study, maternal stature did not alter the effect estimates. The only option we had in our study to investigate the impact of social anchorage was to look at employment status, but this did not modify the results. An indicator for socioeconomic status that often is used is maternal level of education (Luo et al. 2006; Woodruff et al. 2003; Zeka et al. 2008), for which we, unfortunately, had no data. To some extent, this variation is taken into account by using smoking as a proxy because in Sweden the increased risk among women with low education for negative birth outcomes such as LBW, SGA, and PTB could almost entirely be explained by the higher smoking rate among these women (Källén 1999). However, adding a spatial dimension, such as when examining air pollution exposure contrasts, makes the situation more complex, because the covariation between socioeconomic position and exposure to air pollution is not uniform in Scania and may even have different directions between cities (Stroh et al 2005). The biological mechanisms underlying country of origin as a confounder are not clear; hence, more research in the field is needed before it can be clearly stated that it is a true confounder and that we are not concealing the potential risks of air pollution.

Concerning PTB, we observed an opposite trend, suggesting that air pollution might have a small protective effect, which runs counter to earlier results. It might be the case that our air pollution levels underlie air pollution effects on PTB.

A literature review of air pollution studies (Ghosh et al. 2007) indicated that female babies tend to be at greater risk of having LBW, that is, that air pollution effect on LBW is differential with respect to sex. We also had indications in this direction in stratified analyses, with an adjusted OR for baby girls of 1.12 (95% CI, 1.01–1.24) for the highest exposure quartile for NO_x_, and a Wald *p*-value for the interaction term between sex and NO_x_ exposure of 0.09.

Traffic is a major source of local air pollution, and although the exposure assessment methods have become better in recent years, most studies still estimate exposure only at the home address, even if a large proportion of traffic exposure, especially for adults, occurs during commuting time and at the workplace. This is also true in our study. Our study focused on the effects of NO_x_, a gas derived from combustion. NO_x_ levels could respond to levels of particulate matter (PM) ≤ 1 μm in aerodynamic diameter or even ultrafine PM ≤ 0.1 μm in aerodynamic diameter according to previous studies (Arhami et al. 2009; Marconi et al. 2007). NO_x_ is seldom a good marker of PM ≤ 2.5 μm in aerodynamic diameter, which could have other, even nonanthropogenic sources. NO_x_ modeled with a 500 × 500 m resolution is comparably high but cannot pinpoint traffic-generated pollutant peaks. Thus, we also used proximity to road as a more exact source-specific measure of exposure. One limitation with this measure could be that we included only one road (the one with highest traffic density), but the overall low number of high-density roads in our study area could minimize this limitation. Misclassification could also be due to information about mothers’ residency being updated only yearly, and even if we chose the residences they lived in during the longest time period of the pregnancy, we can see that the effects of NO_x_ exposure on the risks of having an SGA baby become statistically significant only in the fully adjusted analysis after excluding mothers who had changed residency during pregnancy. An inherent problem is also that the NO_x_ and traffic exposure categories are strongly correlated with urban–rural contrasts, and there may be other unmeasured confounders that we cannot take into account.

There are several strengths in this study, based primarily on the large amount of accessible data. All mothers have had access to the same maternal health care systems, and all outcomes have been classified using the same standardized protocols. Information about confounders such as smoking has been collected early in pregnancy, and answers could not have been influenced by birth outcomes. The Swedish and regional birth registries contain a large amount of information, which made it possible to adjust for most of the known confounders. We were also able to study newly suggested confounders in detail, such as maternal country of origin.

## Conclusion

Our study, finding no consistent evidence for any negative effects of air pollution on birth outcomes at comparably low exposure levels, clearly points out the need for good exposure estimates and careful control of possible confounding factors, especially those that are linked to socioeconomic and spatial gradients. In our study, parity and maternal country of origin were such factors. Also, it is obvious that large-scale studies are needed to elucidate sex differential effects.

## Figures and Tables

**Table 1:** Background characteristics for birth data.

Characteristic	All (*n* = 81,110)	LBW^a^ (*n* = 2,863)	PTB^b^ (*n* = 4,690)	SGA^c^ (*n* = 7,821)
Maternal age [years (mean ± SD)]	30.4 ± 5.0	30.4 ± 5.5	30.4 ± 5.3	29.8 ± 5.3
Maternal weight [kg (mean ± SD)]	68.0 ± 13.0	65.8 ± 13.8	67.7 ± 13.7	64.1 ± 12.7
Maternal height [cm (mean ± SD)]	167.0 ± 6.4	164.5 ± 6.7	165.4 ± 6.6	164.2 ± 6.5
Infant sex [*n* (%)]				
Boys	41,638 (51.3)	1,432 (50.0)	2,519 (53.7)	4,000 (51.1)
Girls	39,149 (48.3)	1,414 (49.4)	2,161 (46.1)	3,821 (48.9)
Missing	323 (0.4)	17 (0.6)	10 (0.1)	0 (0.0)
Parity [*n* (%)]				
1	38,365 (47.3)	1,687 (58.9)	2,576 (54.9)	4,692 (60.0)
2	26,681 (32.9)	667 (23.3)	1,225 (26.1)	1,902 (24.3)
≥ 3	15,418 (19.0)	488 (17.0)	863 (18.4)	1,188 (15.2)
Missing	646 (0.8)	21 (0.7)	26 (0.6)	39 (0.5)
Smoking in early pregnancy (cigarettes/day) [*n* (%)]				
0	66,086 (81.5)	1,673 (58.4)	3,099 (66.0)	5,611 (71.7)
1–9	6,444 (7.9)	259 (9.0)	354 (7.5)	1,022 (13.1)
≥ 10	2,948 (3.6)	185 (6.5)	205 (4.4)	557 (7.1)
Missing	5,632 (6.9)	746 (26.4)	1,032 (22.0)	631 (8.1)
Maternal prepregnancy body mass index [*n* (%)]				
< 18.5	2,944 (3.6)	142 (5.0)	186 (4.0)	527 (6.7)
18.5–24.9	31,629 (39)	878 (30.7)	1,451 (30.9)	3,112 (39.8)
25–29.9	17,836 (22)	460 (16.1)	897 (19.1)	1,437 (18.4)
≥ 30	7,852 (9.7)	239 (8.3)	445 (9.5)	609 (7.8)
Missing	20,849 (25.7)	1,144 (40)	1,713 (36.5)	2,136 (27.3)
Country of origin [*n* (%)]				
Nordic	63,692 (78.5)	1,879 (65.6)	3,358 (71.6)	5,505 (70.4)
Other	15,921 (19.6)	608 (21.2)	862 (18.4)	2,117 (27.0)
Missing	1,497 (1.8)	376 (13.1)	470 (10.0)	199 (2.5)

a < 2,500 g.

b < 37 gestational weeks.

c < 10th percentile compared with the birth weight distribution for the same gestational week and sex.

**Table 2:** Confounder characteristics and traffic density for the NO_x_ quartiles.

Characteristic	First quartile	Second quartile	Third quartile	Fourth quartile
Maternal age [years (mean ± SD)]	30.4 ± 4.9	30.6 ± 4.8	30.8 ± 5.0	30.0 ± 5.1
Maternal weight [kg (mean ± SD)]	70.2 ± 13.9	68.4 ± 13.0	67.2 ± 12.4	66.6 ± 12.3
Maternal height [cm (mean ± SD)]	166.9 ± 6.2	166.9 ± 6.3	166.5 ± 6.5	166.0 ± 6.6
Infant sex [*n* (%)]				
Boys	10,001 (51.8)	9,747 (51.6)	9,711 (51.2)	9,627 (51.5)
Girls	9,302 (48.2)	9,152 (48.4)	9,248 (48.8)	9,065 (48.5)
Parity [*n* (%)]				
1	7,578 (39.5)	8,301 (44.1)	9,190 (48.6)	10,287 (55.1)
2	7,211 (37.6)	6,896 (36.7)	6,296 (33.3)	5,070 (27.1)
≥ 3	4,376 (22.8)	3,617 (19.2)	3,415 (18.1)	3,329 (17.8)
Smoking in early pregnancy (cigarettes/day) [*n* (%)]				
0	15,593 (80.7)	15,403 (81.5)	15,609 (82.3)	15,327 (81.9)
1–9	1,620 (8.4)	1,551 (8.2)	1,378 (7.3)	1,521 (8.1)
≥ 10	820 (4.2)	611 (3.2)	619 (3.3)	720 (3.8)
Missing	1,279 (6.6)	1,344 (7.1)	1,369 (7.2)	1,145 (6.1)
Maternal prepregnancy body mass index [*n* (%)]				
< 18.5	532 (2.6)	739 (3.7)	750 (3.9)	711 (4.2)
18.5–24.9	7,362 (35.8)	7,699 (39.0)	7,868 (40.9)	6,982 (41.1)
25–29.9	5,041 (24.5)	4,288 (21.7)	4,062 (21.1)	3,534 (20.8)
≥ 30	2,591 (12.6)	1,845 (9.4)	1,581 (8.2)	1,462 (8.6)
Missing	5,054 (24.6)	5,155 (26.1)	4,978 (25.9)	4,291 (25.3)
Maternal country of origin [*n* (%)]				
Nordic	18,727 (91.0)	16,944 (85.9)	14,344 (74.6)	10,894 (64.2)
Other	1,507 (7.3)	2,452 (12.4)	4,543 (23.6)	5,826 (34.3)
Missing	346 (1.7)	330 (1.8)	352 (1.8)	260 (1.5)
Traffic density (cars/min) [*n* (%)]				
No cars	10,605 (55.1)	10,576 (56.2)	8,294 (44.0)	3,672 (19.7)
< 2	7,218 (37.5)	4,607 (24.5)	3,843 (20.4)	3,861 (20.7)
2–5	1,382 (7.2)	3,221 (17.1)	4,292 (22.8)	4,724 (25.3)
6–10	35 (0.2)	407 (2.2)	1,998 (10.6)	2,691 (14.4)
> 10	0 (0.0)	19 (0.1)	437 (2.3)	3,692 (19.8)

**Table 3:** ORs (95% CIs) for birth outcomes by air pollution exposure to NO_x_ and traffic density.

Exposure	Unadjusted	Adjusted^a^	Adjusted^b^	Adjusted^c^
LBW				

NO_x_ (μg/m^3^)				
2.5–8.9	1.00 (Reference)	1.00 (Reference)	1.00 (Reference)	1.00 (Reference)
9.0–14.1	0.86 (0.77–0.96)	0.85 (0.76–0.95)	0.92 (0.82–1.04)	0.90 (0.80–1.01)
14.2–22.6	0.89 (0.80–0.99)	0.84 (0.75–0.94)	0.92 (0.81–1.04)	0.85 (0.75–0.96)
> 22.7	1.04 (0.93–1.16)	0.97 (0.87–1.09)	1.04 (0.92–1.18)	0.93 (0.82–1.06)
Traffic density (cars/min)				
No road	1.00 (Reference)	1.00 (Reference)	1.00 (Reference)	1.00 (Reference)
< 2	1.05 (0.95–1.15)	1.03 (0.93–1.13)	1.06 (0.96–1.18)	1.05 (0.94–1.16)
2–5	1.11 (1.00–1.24)	1.06 (0.95–1.18)	1.08 (0.96–1.21)	1.02 (0.91–1.15)
6–10	1.06 (0.90–1.24)	1.01 (0.86–1.19)	1.07 (0.90–1.27)	0.97 (0.82–1.16)
> 10	1.10 (0.93–1.31)	0.99 (0.83–1.18)	1.14 (0.95–1.37)	1.00 (0.83–1.20)

PTB				

NO_x_ (μg/m^3^)				
2.5–8.9	1.00 (Reference)	1.00 (Reference)	1.00 (Reference)	1.00 (Reference)
9.0–14.1	0.86 (0.79–0.94)	0.85 (0.78–0.93)	0.90 (0.82–0.98)	0.89 (0.81–0.97)
14.2–22.6	0.87 (0.80–0.95)	0.84 (0.77–0.93)	091 (0.83–0.99)	0.87 (0.80–0.96)
> 22.7	0.90 (0.82–0.98)	0.85 (0.77–0.93)	0.91 (0.82–1.00)	0.85 (0.77–0.94)
Traffic density (cars/min)				
No road	1.00 (Reference)	1.00 (Reference)	1.00 (Reference)	1.00 (Reference)
< 2	1.01 (0.94–1.09)	1.00 (0.93–1.08)	1.03 (0.95–1.11)	1.01 (0.94–1.10)
2–5	1.01 (0.92–1.10)	0.98 (0.89–1.06)	1.01 (0.92–1.10)	0.97 (0.88–1.06)
6–10	0.97 (0.85–1.10)	0.93 (0.82–1.06)	0.99 (0.86–1.13)	0.94 (0.82–1.07)
> 10	0.94 (0.81–1.08)	0.87 (0.75–1.00)	0.96 (0.82–1.11)	0.88 (0.76–1.02)

SGA				

NO_x_ (μg/m^3^)				
2.5–8.9	1.00 (Reference)	1.00 (Reference)	1.00 (Reference)	1.00 (Reference)
9.0–14.1	1.03 (0.96–1.10)	1.03 (0.96–1.10)	1.03 (0.96–1.11)	0.99 (0.92–1.06)
14.2–22.6	1.16 (1.08–1.24)	1.13 (1.05–1.21)	1.10 (1.02–1.18)	1.00 (0.94–1.08)
> 22.7	1.37 (1.28–1.47)	1.29 (1.20–1.38)	1.21 (1.12–1.30)	1.07 (0.99–1.15)
Traffic density (cars/min)				
No road	1.00 (Reference)	1.00 (Reference)	1.00 (Reference)	1.00 (Reference)
< 2	1.01 (0.95–1.08)	0.99 (0.93–1.05)	0.98 (0.92–1.05)	0.98 (0.92–1.04)
2–5	1.15 (1.07–1.22)	1.08 (1.01–1.15)	1.09 (1.01–1.16)	1.03 (0.96–1.10)
6–10	1.18 (1.07–1.30)	1.08 (0.98–1.19)	1.08 (0.98–1.19)	0.98 (0.89–1.08)
> 10	1.26 (1.14–1.40)	1.12 (1.01–1.24)	1.19 (1.07–1.33)	1.04 (0.93–1.15)

a Maternal age, smoking, birth year, and sex and parity.

b Maternal age, smoking, birth year, sex, and country of origin.

c Maternal age, smoking, birth year and sex, parity and country of origin.
